# Methyl Jasmonate-Induced Changes of Flavor Profiles During the Processing of Green, Oolong, and Black Tea

**DOI:** 10.3389/fpls.2019.00781

**Published:** 2019-06-14

**Authors:** Jiang Shi, Dongchao Xie, Dandan Qi, Qunhua Peng, Zongmao Chen, Monika Schreiner, Zhi Lin, Susanne Baldermann

**Affiliations:** ^1^Leibniz Institute of Vegetable and Ornamental Crops, Grossbeeren, Germany; ^2^Institute of Nutritional Science, University of Potsdam, Potsdam, Germany; ^3^Key Laboratory of Tea Biology and Resource Utilization, Ministry of Agriculture, Tea Research Institute, Chinese Academy of Agricultural Sciences, Hangzhou, China; ^4^Graduate School of Chinese Academy of Agricultural Sciences, Beijing, China

**Keywords:** methyl jasmonate, aroma quality, volatile compounds, amino acids, tea processing

## Abstract

Tea aroma is one of the most important factors affecting the character and quality of tea. Here we describe the practical application of methyl jasmonate (MeJA) to improve the aroma quality of teas. The changes of selected metabolites during crucial tea processing steps, namely, withering, fixing and rolling, and fermentation, were analyzed. MeJA treatment of tea leaves (12, 24, 48, and 168 h) greatly promotes the aroma quality of green, oolong, and black tea products when comparing with untreated ones (0 h) and as confirmed by sensory evaluation. MeJA modulates the aroma profiles before, during, and after processing. Benzyl alcohol, benzaldehyde, 2-phenylethyl alcohol, phenylacetaldehyde, and trans-2-hexenal increased 1.07- to 3-fold in MeJA-treated fresh leaves and the first two maintained at a higher level in black tea and the last two in green tea. This correlates with a decrease in aromatic amino acids by more than twofold indicating a direct relation to tryptophan- and phenylalanine-derived volatiles. MeJA-treated oolong tea was characterized by a more pleasant aroma. Especially the terpenoids linalool and oxides, geraniol, and carvenol increased by more than twofold.

## Introduction

Tea (*Camellia sinensis* L.) is one of the most traditional non-alcoholic beverages worldwide, which is consumed for its possible health benefits (Preedy, 2013), satisfactory aroma (Kenji and Masuda, 2002; Baldermann et al., 2014; Zheng et al., 2016), and taste (Han et al., 2016). Considering its high economic value, the quality of tea is important for the market value and is mainly defined by its aroma and taste. While phenolic compounds are responsible for the taste, volatile compounds are fundamental for tea aroma (Yang et al., 2013; Baldermann et al., 2014). Undoubtfully, there are quite large variations among different kinds of tea in respect of their volatile compound concentrations and profiles. These variations are largely due to *Camellia* cultivar, region of origin, plucking season, soil, climate, as well as pre- and postharvest treatments (Chen et al., 2010; Lee et al., 2015).

Methyl jasmonate, a well-characterized fatty acid-derived cyclopentanone signaling cue, can induce a series of changes in secondary metabolites in a wide range of plants. MeJA is known to play an important role in promoting the quality of agricultural products, especially in improving the aroma qualities of certain crops, e.g., apple (Rudell et al., 2002; Kondo et al., 2005), banana (Zhao et al., 2010), peach (Meng et al., 2009), strawberry (Ayala-Zavala et al., 2005; Moreno Fde et al., 2010), tea (Shi et al., 2014, 2015), and tomato (Orozco-Cárdenas et al., 2001; Manan et al., 2016). Furthermore, MeJA induces changes in amino acid concentrations and profiles in plants, e.g., tea (Shi et al., 2014, 2015), and grapes (Garde-Cerdan et al., 2016). Amino acids not only affect the taste but also serve as precursors for some volatile compounds such as aldehydes (2-methylbutanal, 3-methylbutanal, 3-methylpropanal, phenylacetaldehyde, etc.) and esters (2-methylpropyl acetate, ethyl 2-methylbutanoate, 2-methylbutyl acetate, benzyl acetate, 2-phenylethyl acetate, etc.) (Gonda et al., 2010; Preedy, 2013; Baldermann et al., 2014; Ho et al., 2015).

Depending on the processing procedure, tea can be divided into green (non-fermented), oolong (semi-fermented), and black (fully fermented) teas. Moreover, the quality of fresh tea leaves partly determines the final tea aroma quality. Normally, the freshly plucked leaves undergo various processing steps that include, depending on the desired final tea product, withering, fixing and rolling, fermentation, and drying (Ho et al., 2008). It is also well known that most of the alcoholic aroma compounds in tea, such as benzyl alcohol, geraniol, linalool and its oxides, and 2-phenylethanol, mainly contribute to the floral, sweet, and fruity aromas found in especially oolong and black teas (Kenji and Masuda, 2002; Baldermann et al., 2014; Zheng et al., 2016). Changes in compound concentrations and profiles occur in fresh tea leaves as well as during the tea processing (Yang et al., 2013; Shi et al., 2014; Lee et al., 2015; Han et al., 2016; Zheng et al., 2016). Our previous study revealed that MeJA activates genes and enzymes involved in numerous processes, e.g., the lipoxygenase (LOX) (green leaf volatiles, GLVs), the volatile terpenoid (TP) biosynthesis, and the shikimate pathway (e.g., volatile phenylpropanoids and benzenoids (VPB) biosynthesis and amino acid metabolism) (Shi et al., 2014, 2015). Considering that amino acids could also be converted to some volatile compounds during tea processing (Horanni and Engelhardt, 2013), it is interesting to profile the dynamic changes of amino acids, as a first step to better understanding their effects on tea aroma and flavor, and thus, overall tea quality.

For this purpose, we applied MeJA and analyzed dynamic changes of volatiles and amino acids in three kinds of tea products (green, oolong, and black tea). Moreover, organoleptic characteristics were determined by sensory evaluation. Here we show for the first time that MeJA treatment modulates the flavor and aroma profiles before, during, and after processing, and such treatment can improve the overall quality of green, oolong, and black tea.

## Materials and Methods

### Chemicals

The following chemicals and reagents were used for the analyses: benzyl alcohol, benzaldehyde, β-cyclocitral, dihydroactinidiolide, eugenol, geraniol, *trans*-2-hexenol, *trans*-2-hexenal, *cis*-3-hexenol, *cis*-3-hexenal, β-ionone, jasmine lactone, linalool, methyl salicylate (MeSA), MeJA, 2-phenylethanol, phenylacetaldehyde, and phenylacetonitrile (Sigma–Aldrich, Germany).

### Tea Plant Materials and MeJA Treatment

*Jinxuan*, a cultivar of the tea plant (*C. sinensis*), planted in Fu’an district, Fujian Province, China was used. Samples were treated and prepared in the spring (March and May) of 2015. Two hectares were selected to apply the field experiment. And 0.4 hectare was evenly sprayed with 0.25% (v/v, every 2.5 ml MeJA pre-dissolved in 25 ml anhydrous ethanol) aqueous solution of MeJA. The specificity was described in (Supplementary Data Sheet 1). The fresh tea leaves (one bud with the two leaves) were plucked by hand after 0, 12, 24, 48, and 168 h after MeJA treatment. The control plants (0 h) were sprayed with distilled water (with the same amount of ethanol) and then processed in the same way as equally to the MeJA-treated samples.

### Fresh Leaves and Tea Processing Samplings

Twenty kilograms of fresh tea leaves was stored directly (0 h) or picked after 12, 24, 48, and 168 h treatment time. A homogeneous aliquot of 1 kg was immediately put into liquid nitrogen for subsequent lyophilization and the rest was used for subsequent tea processing. The tea leaves were subjected to three kinds of tea processing resulting in, namely, green, oolong, and black teas (Table 1); 0 h samples were set as control. The experiments were performed with three biological replicates obtained by pooling 1 kg of fresh leaves or 250 g of each processing step (Table 1).

**TABLE 1:** Sampling-schedule of MeJA-treated tea leaves and crucial steps of tea processing.

**Tea**	**Processing step**	**Samples**
Fresh tea leaves	Fresh tea leaves	
Green tea	Spreading	
	Fixing and rolling	
	Made green tea	
Oolong tea	Green-making 1	Samples were taken after each processing steps for all five sample groups (0, 12, 24, 48, and 168 h)
	Green-making 2	
	Green-making 3	
	Fixing and rolling	
	Made oolong tea		
Black tea	Withering	
	Rolling	
	Fermentation	
	Made black tea	

### Sensory Evaluation and Objective Assessment of Tea Aroma

Small portions of the green, oolong, and black tea products of the MeJA-treated samples were accurately weighed (3.0 g) and placed into 150 ml of boiling water to brew the tea for a period of 5 min. The separated tea leaves were kept in the vessel for sensory evaluation and objective assessment of tea aroma. A well-trained and experienced sensory panel consisting of six members performed a sensory evaluation and objective assessment of tea aroma. The aroma characteristics and quality scores for these made teas were described according to the national standards “Methodology of sensory evaluation of tea” (GB/T 23776-2009).

In this study, the aroma qualities of tea products were evaluated by quality scores using a 100-point scale from 1 to 100. The control sample (0 h) was used as reference and compared to the tea products obtained from tea leaves treated for 12, 24, 48, and 168 h with MeJA. Furthermore, the panelists were required to give extra “descriptive sensory evaluation” taking into account the degree of fresh green, flowery, sweet, and honey-like aroma.

### Volatile Compound Analysis by Gas Chromatograph-Mass Spectrometry (GC-MS)

Stir bar sorptive extraction (GERSTEL-Twisters, PDMS, Gerstel GmbH & Co. KG, Germany) was used to trap the volatile compounds. Ten milligrams (homogeneously ground) lyophilized tea samples were stirred in 5 ml 5% methanol/water solution (v/v) with sodium chloride (saturated) at ambient temperature. The volatile compounds were trapped for exactly 30 min. Each Twister was washed with distilled water, dried, and finally, kept in a GC-Vial under dry conditions prior to analysis. The volatile compounds were analyzed with an Agilent 7890B gas chromatograph (GC) equipped with an Agilent 7010 GC – Triple Quad MS (GC-TQ/MS). The GC was equipped with a BP5MS column (30 m × 250 μm i.d., 0.25 μm; SGE Analytical Sciences) and was operated with the following oven temperature program: 40°C hold for 3 min, increase of 2°C/s until 60°C, and hold for 2 min, then increase of 3°C/min until 180°C, and hold for 10 min isothermally. The carrier gas used was helium and was maintained at a constant flow rate of 1.2 ml/min. The cryofocusing program started at −100°C, the temperature was increased at 12°C/min to 280°C, and then maintained at 280°C for 3 min. Twisters desorption was performed with a Gerstel MPS 2 (multiple purpose sampler) injection system with the following temperature program: starting temperature, 25°C; an increase at 100°C/min until 250°C, and hold for 4 min at 250°C. The MS analysis was carried out in a full-scan mode with a scan range of *m/z* 50−300. The electron impact ionization energy was 70 eV for all measurements. The compounds were identified tentatively by comparing the mass spectra with the Wiley W10N11 and the NIST 14 libraries. In addition, authentic reference compounds were used for the identification of selected compounds (Table 2). Peak areas were obtained after automatic and manual integrations of utilizing mass hunter quantitative and qualitative analyses (Version B. 07.00; Agilent Technologies). Heat maps were prepared by Statistica (version 12.0; Dell Co.) and Excel (version 2016), and used for visualization. The GC-TQ/MS analysis was performed using three biological replicates.

**TABLE 2:** Key volatile compounds and the odor description.

**No.**	**Volatile compound**	**Subclass**	**Subclass**	**Origins (pathways)**	**Odor description**
1	*cis*-3-Hexenol^s^	Alcohols	GLVs	LOX	Green
2	*trans*-2-Hexenol^s^		GLVs	LOX	Green grassy
3	Benzyl alcohol^s^		VPBs	Shikimate	Sweet, floral
4	2-Phenyl alcohol^s^		VPBs	Shikimate	Floral
5	Linalool^s^		TPs	Terpenoids	Sweet floral
6	Geraniol^s^		TPs	Terpenoids	Rose-like, floral
7	Linalool oxide 2		TPs	Terpenoids	Sweet floral
8	Linalool oxide 3		TPs	Terpenoids	Sweet floral
9	Carvenol		TPs	Terpenoids	–
10	Nerolidol		TPs	Terpenoids	Floral
11	Eugenol^s^		VPBs	Terpenoids	Spicy
12	*cis*-3-Hexenal^s^	Aldehydes	GLVs	LOX	Green, earthy
13	*trans*-2-Hexenal^s^		GLVs	LOX	Fruity
14	Nonadienal		GLVs	LOX	Green, cucumber-like
15	Heptanal		GLVs	LOX	Burnt plastic, smoky
16	Benzaldehyde^s^		VPBs	Shikimate	Honey, floral
17	Phenylacetaldehyde^s^		VPBs	Shikimate	Honey-like, sweet
18	Octanal		GLVs	LOX	Fat, lemon-like, green
19	Decanal		GLVs	LOX	Waxy
20	Nonanal		GLVs	LOX	Citrus fruity
21	β-Cyclocitral^s^		TPs	Terpenoids	Mild green, minty, fruity
22	Methyl salicylate^s^	Esters	VPBs	Shikimate	Nutty, floral
23	Methyl jasmonate^s^		GLVs	LOX	Floral
24	Jasmine lactone^s^	Ketones	GLVs	LOX	Jasmine-like, floral
25	Dihydroactinidiolide^s^		TPs	Terpenoids	Sweet
26	β-Ionone^s^		TPs	Terpenoids	Rose floral
27	1-Octen-3-one		GLVs	LOX	Mushroom-like
28	Phenylacetonitrile^s^	Others	VPBs	Shikimate	–
29	β-Myrcene		TPs	Terpenoids	–
30	Limonene		TPs	Terpenoids	Fruity
31	Indole		Others	Shikimate	Animal-like

*^s^Volatile compounds identified by reference compounds. GLVs, green leaf volatiles; TPs, volatile terpenoids; VPBs, volatile phenylpropanoids and benzenoids; LOX, lipoxygenase pathway; –, no clear odor description from other literatures.*

### Amino Acids Analysis by a MembraPure Amino Acid Analyzer

Free amino acid analysis was performed using 5 mg lyophilized tea sample. The analysis was performed in biological triplicates. The amino acids were extracted by sonication with 250 μl methanol (MeOH, 70% pH 2, kept at 4°C) for 15 min on ice. After centrifugation (5 min, 4000 *g*, at 4°C), the supernatant was collected in a 2 ml Eppendorf tube. The residue was re-extracted by 100 μl methanolic extraction solvent and sonication for 10 min. The combined supernatants were adjusted to 400 and 100 μl of precipitation solution (MembraPure GmbH, Germany) were added before being incubated for 1 h at 4°C. Prior to analysis, the extract was filtered through a 0.22 μm cellulose membrane. The amino acid analysis was carried out with a MembraPure Amino Acid Analyzer (MembraPure GmbH, Hennigsdorf, Germany) according to manufacturer’s instructions. The data were analyzed with the Chromatography Data Handling System (Amino peak v. 2.36, MembraPure GmbH, Germany) and then processed by Excel 2016 (Microsoft). Finally, statistical analysis was performed by Statistica (version 12.0; Dell Co.).

### Statistical Analysis

The statistical analysis of all the data sets (one-way ANOVA, Tukey’s HSD *post hoc* test, *p* ≤ 0.05) was performed with Statistica (version 12.0; Dell Co.). Principle component analysis (PCA) and factor analysis were also performed with Statistica and heat maps were generated using Heml (version 1.0).

## Results and Discussion

### Descriptive Sensory Characterization of the Green, Oolong, and Black Tea Produced From MeJA-Treated Leaf Samples

All the panelists invited were trained for several years in tea aroma quality sensory evaluation including odor description and intensity. A descriptive sensory evaluation was carried out by the panel using the following attributes: fresh green, flowery, sweet, and honey-like (an intensity scale from 80 to 100 was applied for which a higher score means a stronger attribute expression). The overall quality score of the MeJA-treated teas was mostly rated higher compared to the untreated control (0 h), except for the samples treated for 168 h and processed to green or black tea (Table 3). The findings show that the aroma quality of green, oolong, and black tea products can be improved by MeJA treatment as well as the duration of adaptation time after MeJA treatment.

**TABLE 3:** Sensory assessment of tea prepared from MeJA-treated fresh tea leaves.

**Treatment time (h)**	**Green tea**		**Oolong tea**		**Black tea**	
	**Score**	**Odor**	**Score**	**Odor**	**Score**	**Odor**
0^a^	88.6±2.5	–	88.2±2.5	–	88.1±2.74	–
12	$90\pm 2.83^{*}$	Slightly flowery	$94.2\pm 2.48^{**}$	Strong flowery, honey-like	$91\pm 2.35^{**}$	Sweet, honey-like
24	$94.8\pm 1.3^{**}$		$90.8\pm 2.86^{*}$		$91.8\pm 2.55^{**}$	
48	89.2±1.92	–	$93.1\pm 1.83^{**}$		$91.4\pm 1.34^{**}$	
168	88.5±2.03	–	$94.8\pm 2.09^{**}$		87.1±2.16	–

^*^p < 0.05, ^∗∗^*p* < 0.01; a higher score represents a higher aroma quality. ^a^0 h samples were the control ones which were processed as following: first one bud with two leaves were picked before MeJA treatment, then followed with the same tea processing procedure as the other samples into made green tea, oolong tea, and black tea.

In detail, the changes obtained in flavor profiles and quality of green tea are time dependent, especially the tea products prepared from leaves treated with MeJA for 12 and 24 h developed a slightly flowery aroma. Note that the tea quality grade is rated higher when a slightly flowery aroma is present (Rawat et al., 2007; Jumtee et al., 2011; Shi et al., 2015). In our study, not only green tea, but also the quality of oolong tea was improved. Strong flowery and honey-like aroma was notably in teas obtained after 12 h of MeJA treatment and onward. In China, as well as in other Asian countries, oolong tea is very popular (Zhu et al., 2015). In the case of the black tea, the evaluation showed that between 12 and 48 h MeJA-treated leaves could be suitable for making black tea with a honey-sweet aroma. Since all these changes in aroma characteristics in green, oolong, and black tea are positively associated with improved tea quality, pre-harvest MeJA treatment seems to be an effective and potential alternative treatment method to promote overall tea quality.

### Aroma Profiling of Tea Products Produced From MeJA-Treated Leaf Samples

**FIGURE 1:**
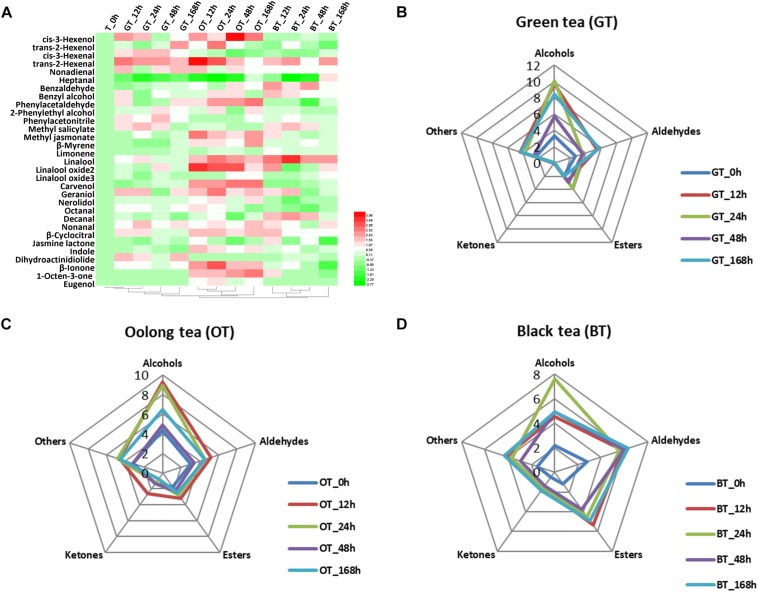
Key volatile compounds in tea products produced from MeJA-treated fresh tea leaves. **(A)** Differentially abundant flavor volatile compounds in three kinds of tea products. Radar maps of differentially abundant volatile compounds in **(B)** green tea (GT), **(C)** oolong tea (OT), and **(D)** black tea (BT) prepared from MeJA-treated fresh tea leaves (FL). Green tea products prepared from MeJA-treated fresh tea leaves after 0, 12, 24, 48, and 168 h. Sampling times of oolong tea and black tea were identical. Color scale represents fold changes, in general “white” means “no change,” “red” means “increase,” and “green” means “decrease.”

The volatile compound profiles of green, oolong, and black teas prepared from MeJA-treated leaf samples after five different treatment adaptation durations were analyzed. A factor analysis revealed a difference in 31 flavor volatile compounds in tea products, which were then further analyzed (Figure 1A). These volatile compounds are divided into alcohols, aldehydes, esters, ketones, and others (Table 2). All of these volatile compounds contribute an odor that affects the overall tea aroma (Table 2). Zheng et al. summarized the most characterized aroma in different kinds of tea and reported that green and oolong tea flavor is mainly characterized by alcohols. Additionally, aldehydes, esters, and other compounds (e.g., phenyl acetonitrile, β-myrcene, limonene, and indole) also contribute to the aroma. In this study, rising relative levels of volatile compounds after MeJA treatment were detected in all three kinds of tea products likely also contributing a higher overall quality score of the tea products. Moreover, these changes were also time dependent, e.g., highest relative abundances of volatile compounds were found 12 h after MeJA treatment in green and oolong, but after 24 h in black tea (Figures 1B–D). The alcohols and aldehydes are known to be major flavor compounds in tea (Table 2).

**FIGURE 2:**
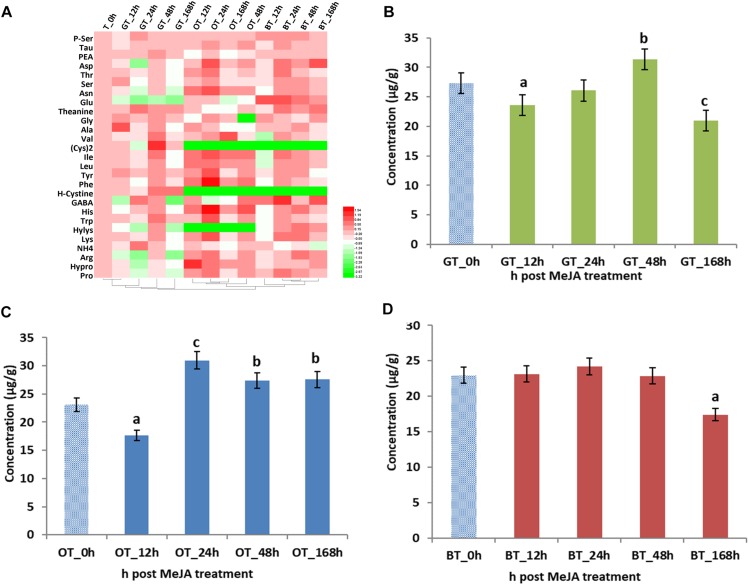
Amino acids (AAs) in processed tea products prepared from MeJA-treated fresh tea leaves. Data represent the mean value ± standard deviation of three independent samples. Means distinguished with different letters are significantly different from control (one-way ANOVA, *p* < 0.05). **(A)** Differential AAs in three kinds of tea products. Total contents of AAs in in **(B)** green tea (GT), **(C)** oolong tea (OT), and **(D)** black tea (BT) prepared from MeJA-treated fresh tea leaves. Color scale represents fold changes, in general “white” means “no change,” “red” means “increase,” and “green” means “decrease.”

Volatile compounds that contribute to tea aroma are mainly derived from the LOX, the terpenoid, and the shikimate pathway (Table 2). In addition, amino acids derived from shikimate pathway, especially tryptophan and phenylalanine, are also important as tea aroma precursor (Ho et al., 2015). Thus, we investigated the changes of 27 amino acids and their abundances are summarized in Figure 2. Previous studies revealed that MeJA treatment greatly changed the amino acid content in grape (e.g., histidine, serine, tryptophan, tyrosine, asparagine, methionine, lysine, and especially, phenylalanine) (Garde-Cerdan et al., 2016). In our study, the changes in amino acid abundance depended on the type of processing, e.g., increased concentrations in the total amino acid concentration were found after 48 and 24 h in green and oolong tea, respectively. However, no increase was found for the black tea. Finally, lower amino acid concentrations were found after 12 h in green and oolong tea as well as after 168 h in green and black tea. Thus, it seems to be the best to harvest all three kinds of tea after 24 h post MeJA treatment, respectively.

#### Changes in Green Tea Products

The flavor of green tea is strongly impacted by alcohols and aldehydes (Figure 1B). Alcoholic aroma compounds were massively accumulated in green teas prepared from 12, 24, and 168 h MeJA-treated raw material (Figures 1A,B). *cis*-3-Hexenol, benzyl alcohol, and geraniol increased after MeJA treatment and maximum levels were reached after 12 h. *cis*-3-Hexenol is recognized as a leafy alcohol that contributes to the green and fresh aroma in green tea (Ho et al., 2008). Benzyl alcohol contributes to flowery attribute in teas (Ho et al., 2015). Geraniol is one of the most important volatile compounds in all teas (Horanni and Engelhardt, 2013). According to Han et al. (2016), the levels of linalool and geraniol decrease during tea processing. Interestingly, in our study, the relative content of geraniol increased more than twofold compared to the control. This increase shows the potential of MeJA treatment to enhance this key flavor in green tea products. According to Zheng et al. (2016), VPBs are important for flavor in both oolong and black tea. Benzyl alcohol and phenylacetaldehyde were higher in green tea after 12 h of MeJA treatment. Generally, this kind of aroma is much more pronounced in steamed green tea (Japanese green tea) and not in panning green tea (Chinese green tea) (Ho et al., 2015). Based on the flavor character of these key compounds, their increase can explain the overall flavor change and the higher sensory quality scores (Table 1).

Considering the importance of the contribution of amino acids to the overall quality of tea (Kaneko et al., 2006), the different abundances of amino acids were also analyzed. In tea samples after 12, 24, and 168 h of MeJA treatment, lower concentrations of amino acids were detected (Figure 2B). This decrease can be explained by lower concentrations of glutamate (Glu) and GABA (Figure 2A). GABA is an important amino acid in tea since health benefits for humans have been reported such as relaxation and improved immunity (Gilliham and Tyerman, 2016). With Glu decreasing and GABA increasing to a maximum value in the 24 h sample, we speculate that MeJA impacts the conversion step from Glu to GABA since it is already known that Glu is easily converted to GABA by the Glu decarboxylase (GAD) and that this gene is upregulated by MeJA. Hence, increased levels of GABA could positively impact on tea quality. As the precursor for VPBs (Peled-Zehavi et al., 2015; Cheng et al., 2016), the decrease of phenylalanine (Phe) after 12 h post MeJA treatment potentially contributes to the increase of phenylactealdehyde and benzyl alcohol in green tea.

Thus, more intensive aroma in green teas induced by a slightly flowery aroma obtained after MeJA treatment can partly be explained by the direct changes observed for the key volatile compounds, namely *cis*-3-hexenol, benzyl alcohol, and geraniol. Moreover, the changes in amino acid abundances seem also to contribute to improved overall quality of green tea.

#### Changes in Oolong Tea Products

Oolong tea products derived from MeJA-treated leaf samples after 12 h contain twofold higher relative amounts of volatile compounds compared to the control (Figure 1C). In MeJA-treated oolong tea samples, important flavor compounds were affected (Figure 1A). Among these were alcoholic compounds, such as *cis*-3-hexenol, benzyl alcohol, linalool and its oxide 2, carvenol, geraniol, and nerolidol – all of which increased compared with the control group. Moreover, aldehydes, such as *trans*-2-hexenal, phenylacetaldehyde, and β-cyclocitral, were more abundant (Figure 1A). Generally, in oolong tea, nerolidol, indole, linalool, benzaldehyde, as well as linalool oxide 1 are the most abundant volatile compounds. The difference between our results and those of other studies is most likely due to the effect of MeJA treatment. For example, according to Sheibani et al. (2016a), nerolidol, *trans*-2-hexenal, benzaldehyde, indole, geraniol, benzyl alcohol, linalool oxide, and *cis*-jasmone are all important volatile compounds that contribute both to preferred aroma, and thus, priced oolong tea. In our study, a twofold increase in *trans*-2-hexenal, linalool oxide 2, geraniol, and indole promoted a strong flowery, honey-like aroma in oolong tea (Table 2). Finally, the other important improvement to overall tea aroma quality was due to MeJA treatment and the therefrom increasing relative amounts of MeJA itself that has been reported to produce a floral and sweet aroma in oolong tea (Kenji and Masuda, 2002).

Total amino acid content was also significantly influenced by MeJA treatment (Figure 2C). Lower concentrations were found in the 12 h MeJA-treated samples, while higher concentrations were observed in 24, 48, and 168 h samples (Figure 2C). In addition, a conversion has been described by Gonda et al. (2010) who showed the catabolism of amino acids (e.g., L-isoleucine, L-leucine, L-valine, L-methionine, and L-phenylalanine) into aroma volatile compounds (methyl butanal, methyl propanal, and VPBs) via a transamination mechanism (Gonda et al., 2010). Indole is the crucial intermediate in the biosynthesis of Trp (Zeng et al., 2016) and indole efficiently and rapidly responds to exogenous stimuli such as wounding, insects, and MeJA (Erb et al., 2015). Moreover, it is clear that jasmonate signaling is triggered by MeJA in tea leaves (Meng et al., 2009) and that the accumulation of indole could be the result of a 1.5-fold increased in Trp biosynthesis (24 and 48 h after MeJA treatment). Thus, the strong floral aroma obtained after MeJA treatment in oolong tea can partly be explained by changes observed for the key volatile compounds, namely, geraniol, linalool and oxides, β-cyclocitral, *trans*-2-hexenal, MeJA, benzyl alcohol, and phenylacetaldehyde. In addition, changes in Phe and Trp levels might be linked to the formation of VPBs and indole, and thus, likely contribute to the improved *aroma* of this tea type.

#### Changes in Black Tea Products

Alcohols, aldehydes, and esters are highly abundant in black tea products (Figure 1D), which is in accordance with previous studies (Kenji and Masuda, 2002; Ho et al., 2008). Alcoholic volatile compounds were observed predominantly in the 24 h MeJA-treated samples (Figure 1D), e.g., benzyl alcohol and geraniol increased by 1.5-fold and linalool increased by threefold in relative abundance. Such increases are in accordance with Zheng et al. (2016) who have summarized the top characterized aroma compounds in black tea to be *trans*-2-hexenal, geraniol, linalool, linalool oxide 2, benzaldehyde, linalool oxide 1, and MeSA. Of note is that benzyl alcohol, geraniol, and linalool have all been proven to have a floral smell (Table 2) and the promotion of these floral compounds is fundamental for the improved aroma of black tea observed in this study (Table 3). In addition, *trans*-2-hexenal, and β-cyclocitral were also increased in black tea products (12 h). This finding is in accordance with our previous study in which we demonstrated that MeJA treatment promotes α-linolenic acid metabolism, carotenoids metabolism, and terpenoids backbone biosynthesis (Shi et al., 2014, 2015).

The total amino acid abundance in the 12, 24, and 48 h MeJA-treated samples did not change, while a decrease in the 168 h samples was detected (Figure 2D). However, individual amino acids behaved differently, especially in the 12 h samples. Most of the amino acids decreased, except for Glu and GABA, which increased by twofold. To date, only a very few publications discuss the possibility of a transformation of branched-chain and aromatic amino acids into short-chain volatile compounds, e.g., leucine into 3-methyl butanal, isoleucine into 2-methyl butanol, and valine into 2-methyl propanol (Ho et al., 2015). However, in our study, we only find a difference in the abundance of some fatty acid derived aroma, e.g., decanal, octanal, nonanal. Finally, in contrast to oolong tea, the Phe concentration decreased in 12 h MeJA-treated black tea samples and the benzyl alcohol level increased.

In summary, the improved quality of the black tea products is mainly related to the changes in the abundance of linalool, benzyl alcohol, and geraniol as well as the changes of the putative aroma precursors present, e.g., Phe.

### Flavor Volatile Compounds and Amino Acids Changes in MeJA-Treated Fresh Tea Leaves

**FIGURE 3:**
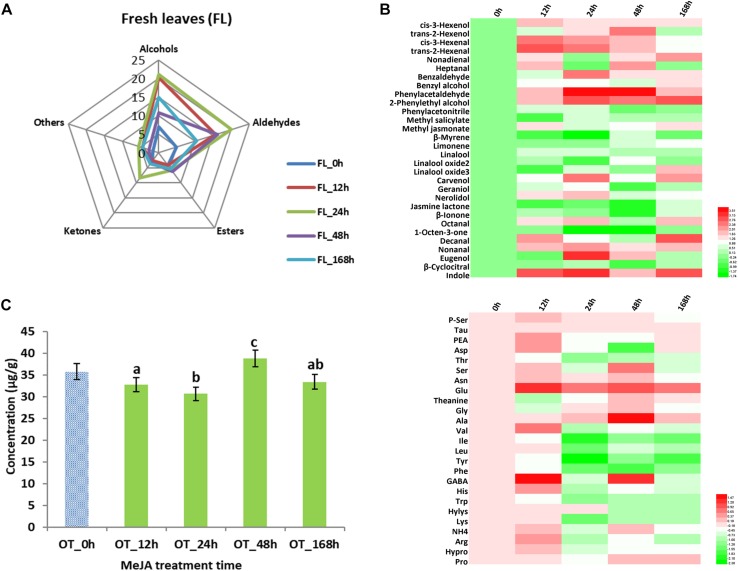
Key volatile compounds and amino acids (AAs) configurations in MeJA-treated fresh tea leaves. **(A)** Radar map of differential volatile compounds in MeJA-treated fresh tea leaves. **(B)** Differential volatile compounds in MeJA-treated fresh tea leaves. **(C)** Total contents of AAs in MeJA-treated fresh tea leaves. **(D)** Differential AAs in MeJA-treated fresh tea leaves. Color scale represents fold changes, in general “white” means “no change,” “red” means “increase,” and “green” means “decrease.”

In addition to studying changes in tea products, volatile compounds and amino acid analyses were carried out in fresh tea leaves (Figure 3). We found that 31 volatile compounds (Table 2) showed changed abundance and that the 24 h samples had the highest level of alcohols, aldehydes, and ketones (Figure 3A). For example, higher contents of hexenols and hexenals were detected (Figure 3B) – both of which are involved in conferring the basis of the grassy green smell of fresh tea leaves (Table 2). Our previous results demonstrated that alcohol dehydrogenase (ADH) expression improved after MeJA treatment (Meng et al., 2009), and this is known to accelerate the conversion of hexenols into hexenals (Ho et al., 2008; Baldermann et al., 2014; Zheng et al., 2016). Similar to a study that described changes in volatile profiles of tomatoes treated with MeJA (Errard et al., 2015), many volatile compounds showed differential abundance, especially nonadienal, octanal, nonanal, heptanal, and decanal. Interestingly, all increased more than twofold in samples treated with MeJA after 12 and 24 h (Table 2).

Volatile terpenoids comprise a major group of volatile compounds in tea plants, and most of them give rise to a floral smell, e.g., linalool and its oxides (sweet floral), geraniol (rose like), and nerolidol (floral) (Baldermann et al., 2014; Zheng et al., 2016). We found a slight decline of β-myrcene, limonene, and linalool oxides in 12 and 24 h MeJA-treated samples. In contrast, linalool, carvenol, and geraniol showed a slight increase in 12 h MeJA-treated fresh leaves, while nerolidol showed a significant increase in both 12 and 24 h MeJA-treated fresh leaves. Based on our previous results, we therefore hypothesize that the accumulation of the precursor geranyl pyrophosphate (GPP) is related to TPs biosynthesis (Meng et al., 2009) and is finally converted to linalool, nerolidol, carvenol, and geraniol during tea processing. This hypothesis could partly explain why slight changes of TPs occurred in fresh tea leaves, while significant changes appeared in tea products.

Previously, MeJA was shown to upregulate Phe related metabolism pathways in tea plants (Shi et al., 2014, 2015). This is in accordance with this study in which we detected a more than twofold increase in benzaldehyde and 2-phenylethyl alcohol as well as a more than threefold increase in phenylacetaldehyde and eugenol in 24 h MeJA-treated samples. As mentioned in Table 2, the VPBs and eugenol can contribute to a flowery and a spicy smell, respectively. The storage of volatile glycosidic precursors in leaves, e.g., tea (Wang et al., 2000), fruits, and flowers (Peled-Zehavi et al., 2015; Cheng et al., 2016), is well documented and the hydrolysis of such precursors into volatile compound has also been studied (Wang et al., 2000; Baba and Kumazawa, 2014). According to the recent literature (Tamogami et al., 2016), the identification of glycosidic volatile compounds during tea processing clearly highlights the importance of glycosidic precursors as a source of a free form volatile compounds. Although differential abundance of some UDP-glycosyltranferases was confirmed in a previous study, it is still unclear how MeJA treatment could play role on their dynamics in fresh tea leaves.

The total amount of amino acids in the fresh leaves rose, especially in samples treated with MeJA after 24 and 48 h (Figure 3C). Due to the complexity of amino acid biotransformation pathways, it is difficult to acquire a thorough view of the pertaining changes. The accumulation of jasmonic acid-isoleucine (JA-Ile) conjugate, which can activate the JA pathway in plants (Liechti and Farmer, 2002; Santino et al., 2013), might cause a big decline of Ile as seen in a more than twofold decrease in 24 h MeJA-treated samples in this study. Moreover, we found that Trp, which is related to indole synthesis, and Phe, which is related to VPBs (Gonda et al., 2010; Zheng et al., 2016), were also decreased significantly in samples treated with MeJA for 24 h (Figure 3D).

### Flavor Volatile Compounds and Amino Acids Changes During Tea Processing

Tea processing is known to greatly affect the flavor compounds and has been extensively discussed in the literature (Katsuno et al., 2014; Sheibani et al., 2016b; Wang et al., 2016). In general, most of the aroma compounds undergo chemical transformations during tea processing and are derived from, e.g., unsaturated fatty acids (GLVs), amino acids (short aldehydes), carotenoids (β-cyclocitral and β-ionone), and glycosides (TPs and VPBs) (Wang et al., 2000; Baba and Kumazawa, 2014).

### Differential Flavor Compounds in Green Tea Processing

**FIGURE 4:**
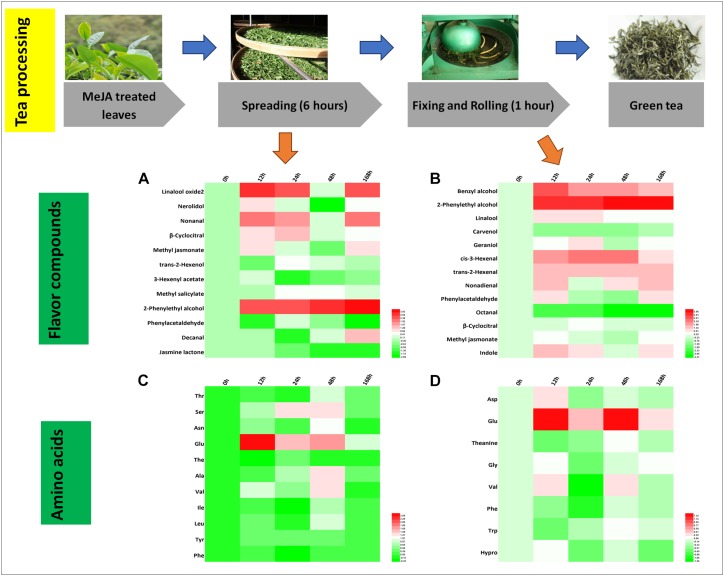
Characteristic compounds changed during green tea processing. Differential volatile compounds during **(A)** spreading as well as **(B)** fixing and rolling processing. Differential AAs during **(C)** spreading as well as **(D)** fixing and rolling processing. Color scale represents fold changes, in general “white” means “no change,” “red” means “increase,” and “green” means “decrease.”

Green tea is a non-fermented tea and its processing mainly consists of spreading, fixing and rolling, and drying. We found that 12 and 13 crucial aroma compounds were subjected to changes during the spreading and the rolling process, respectively (Figures 4A,B).

During the spreading process, the abundance of floral compounds rose by two to threefold for three alcohols (linalool oxide 2, nerolidol, and 2-phenylethyl alcohol), two aldehydes (nonanal and β-cyclocitral), and one ester (MeJA). Four of them (linalool oxide 2, nerolidol, β-cyclocitral, and 2-phenylethyl alcohol) remained at higher levels throughout all the time points and two of them (nonanal and MeJA) decrease in both the 24 and the 168 h samples. The amino acid concentrations and profile changed slightly during the spreading process (Figure 4C), and only Glu, a potential precursor of GABA (Gilliham and Tyerman, 2016), was increased significantly in the MeJA-treated samples.

During the rolling process, volatile compounds are accumulated. In the present study, *cis*-3-hexenal (contributing to a fresh smell) and *trans*-2-hexenal (contributing to a fruity smell), two very important flavor compounds, increased in abundance. In the 12 and 24 h samples, benzyl alcohol, 2-phenylethyl alcohol, linalool, nonadienal, and phenylacetaldehyde relatively increased up to twofold (Figure 4B). Phe declined during this process (Figure 4D), and according to others (Orlova et al., 2006; Tan et al., 2016), the degradation of Phe has been reported to promote the formation of VPBs.

In stark contrast to the fact that higher relative amounts of hexenols and hexenals are stored in MeJA-treated fresh leaves, we only found *trans*-2-hexenol with a lower abundance in the spreading samples (Figure 4A) concluding that MeJA additionally induces the synthesis of geraniol (Figures 3B, 4B). A previous study has shown that linalool and its oxide concentrations decrease during the rolling process. However, this is in contrast to our study in which higher relative abundances of these compounds were detected in MeJA-treated samples. One study has posited the hypothesis that these volatile compounds are derived from glycosidically bound precursors or oxidized and esterized intermediates (Cui et al., 2016). Such modification reactions could be triggered by MeJA treatment activating a series of differential expression of genes related to the LOX pathway (e.g., LOX and O-methyltransferase), TPs pathway (e.g., GPP synthase and UDP-glycosyltransferase), and shikimate pathway (e.g., UDP-glycosyltransferase and cytochrome P450s) (Cui et al., 2016; Tamogami et al., 2016; Cna’ani et al., 2017). These glycosides could be released after the rolling process. In this context, we found higher relative levels of 2-phenylethyl alcohol and benzyl alcohol in all the fixing and rolling samples (Figure 4B) that are both involved in creating the slightly flowery aroma and from which changes remained until the final green tea product was processed. The slightly flowery aroma in green tea products can be attributed to the increase in floral compounds mainly derived from amino acids transformation and release from precursors.

### Differential Flavor Compounds in Oolong Tea Processing

**FIGURE 5:**
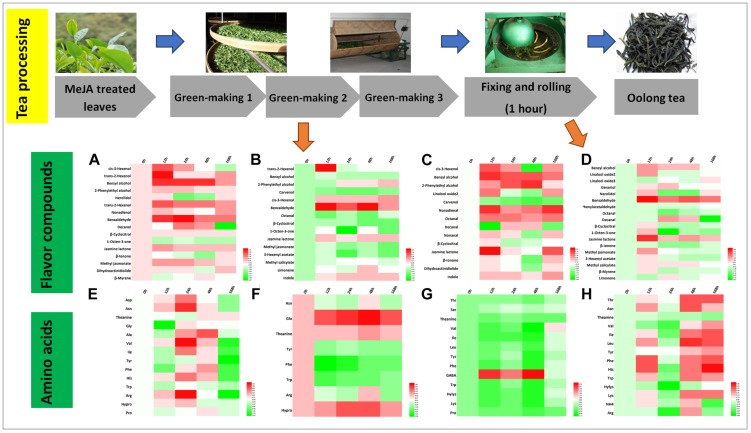
Characteristic compounds changed during oolong tea processing. Differential volatile compounds during **(A)** green-making 1, **(B)** green-making 2, **(C)** green-making 3, and **(D)** fixing and rolling processing. Differential AAs in **(E)** green-making 1, **(F)** green-making 2, **(G)** green-making 3, and **(H)** fixing and rolling processing. Color scale represents fold changes, in general “white” means “no change,” “red” means “increase,” and “green” means “decrease.”

Oolong tea is a semi-fermented tea that originated from China, and its processing includes green-making, fixing and rolling, and drying (Lin et al., 2015). Changes in volatile compounds and amino acids during crucial processing steps, i.e., green-making and rolling, were investigated (Figures 5A–D). During the first green-making stage, *trans*-2-hexenol and *cis*-3-hexenol were associated with a fresh smell and benzyl alcohol and 2-phenylethyl alcohol conferred a sweet and flowery aroma (Table 2) that relatively increased two to threefold in the 12 and 24 h samples, respectively. The second green-making stage led to a threefold relative increase in benzaldehyde in MeJA-treated samples. Moreover, jasmine lactone (jasmine-like, floral), *cis*-3-hexenal (green, earthy), and indole (animal-like) increased (1.5-2.5-fold) in all MeJA-treated samples (Figure 5B). After the last green-making stage, the relative abundance of benzyl alcohol, 2-phenylethyl alcohol, and linalool oxides as well as nonadienal, octanal, and β-cyclocitral increased. Most of the amino acids (e.g., Asn, theanine, Tyr, Phe, Trp, Ile, His, Val, Leu) increased at the beginning but decreased significantly at the end of green-making process (Figures 5E–G). During this processing stage, levels of Phe and Trp greatly decreased and led to the formation of VPBs and indole, respectively (Figures 5E–H) (Ho et al., 2008). Finally, levels of GABA in tea samples treated with MeJA after 12, 24, and 48 h almost increased threefold (Figure 5G).

The rolling step is important in the production of oolong tea during which huge amounts of volatile compounds are formed and released (Cho et al., 2007). In this study, in the oolong tea samples of 12 h post MeJA treatment, benzyl alcohol, geraniol, linalool, benzaldehyde, nerolidol, β-cyclocitral, jasmone lactone, and 1-octen-3-one relatively increased (Figure 5D). All these compounds contribute to the flowery and sweet aroma of oolong tea (Table 2). Further, the hexenals and hexenols can be converted to each other, and the increase in relative levels of *cis*-3-hexenyl acetate after the rolling processing step contributes to the formation of GLVs in oolong tea products. In contrast to green tea, monoterpene alcohols (linalool and oxides, nerolidol, and geraniol) nearly disappeared during the green-making (Figures 5A–C) in oolong tea. The lower relative abundance of these monoterpene alcohols in fresh tea leaves and green-making samples could be due to glycosylation as previously reported in tea plants (Venkata and Prakash, 2011; Tarun et al., 2016). Moreover, during the rolling step, the cell walls and membranes are damaged that may lead to the release of hydrolysis enzymes that form free monoterpene alcohols. Such an enzymatic reaction would explain the higher levels of geraniol, nerolidol, and linalool oxide 2 found afterward in this study.

During the processing of oolong tea, volatile compounds originated from three predominant pathways, namely, the LOX (hexenols, hexenal, and MeJA), the terpenoids biosynthesis (linalool and oxides, nerolidol, and geraniol), and shikimate metabolism (VPBs and indole) showed differential abundance. However, the mechanisms leading to the release of flavor compound are less clear and therefore need further research.

### Differential Flavor Compounds in Black Tea Processing

**FIGURE 6:**
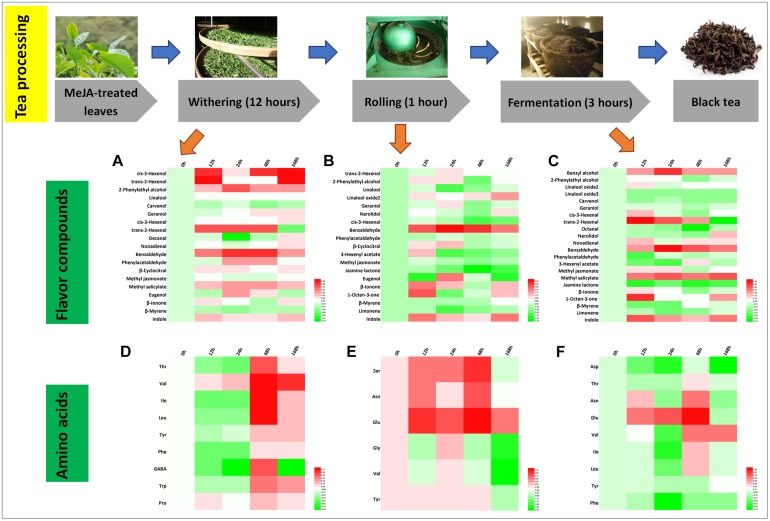
Characteristic compounds changed during black tea processing. Differential flavor volatile compounds during **(A)** withering, **(B)** rolling, and **(C)** fermentation processing. Differential AAs during **(D)** withering, **(E)** rolling, and **(F)** fermentation processing. Color scale represents fold changes, in general “white” means “no change,” “red” means “increase,” and “green” means “decrease.”

Black tea is popular all over the world and is a total fermented tea product. The processing of black tea includes withering, rolling, fermentation, and drying (Horanni and Engelhardt, 2013; Shi et al., 2015). Changes of 19 flavor aroma compounds were identified during the processing steps (Figures 6A–C). It is notable that relative hexenol levels increased significantly, which is likely due to the activation of the jasmonic acid (JA) pathway (Figures 6A–C; Liechti and Farmer, 2002; Shi et al., 2015). As expected, the green smell in black tea declined (*cis*-3-hexenol, *trans*-2-hexenol, and *cis*-3-hexenal), whereas the fruity aroma increased (*trans*-2-hexenal, higher relative abundance in withering and fermentation samples) (Tables 2, 3). During the rolling process of black tea, volatile compounds stored during the withering process are released. The enzymes in the tea leaves are thought to still be active (Katsuno et al., 2014; Shi et al., 2014), resulting in activating the plant’s secondary metabolisms, including volatile compound biosynthesis during the withering and rolling steps (Tan et al., 2016). The rolling step will break cell walls and membranes, resulting in the release of volatile compounds. In addition, the fermentation step is very important in the processing of black tea during which oxidation occurs (Venkata and Prakash, 2011; Tarun et al., 2016). This step lasted for 3 h and many crucial volatile compound changes occurred during this time, e.g., benzyl alcohol, benzaldehyde, MeSA, jasmine lactone, and *trans*-2-hexenal, most of which increased (Figures 6A–C). In a previous study, they monitored the fluctuation of compounds during fermentation and found that the 24 h withering sample contained the maximum level of floral volatile components, namely, phenylacetaldehyde and 2-phenylethyl alcohol. These changes can be linked to lower Phe concentrations (Figure 6D). The more than threefold increase in several amino acids, such as Leu, Ile, and Val (Figures 6E,F), could contribute to the accumulation of key aldehydes, e.g., butanal or propanal (Ho et al., 2015).

Finally, during processing, volatile compounds are released from their glycosidic precursors, e.g., linalool, which is found in high amounts in black tea products. Song et al. (2018) reviewed the function of glycosidically bound volatiles in plants and discussed that these compounds are stable during storage. These compounds can be released during tea manufacturing and likely also contribute to the changes observed in this study. According to Jing et al. (2019), the volatile glycoside [(Z)-3-hexenyl glucoside] is a defense compound responding to (a)biotic stress. In our study, MeJA treatment mimics a (a)biotic attack and enforces storage of glycosidically bound volatiles and thus the defense capability. Interestingly, indole significantly increased during black tea processing. Zeng et al. (2016) demonstrated that due to continuous wounding stress, indole production is induced in oolong tea. In black tea, indole is derived from indole-3-glycerol phosphate (IGP). The high relative indole level found in our tea samples is therefore the result of both increased biosynthesis along with increased release from its glycosidic precursors. Thus, in summary, MeJA treatment resulted in an improvement in the intensity of the black tea aroma due to increased levels of key volatile compounds, predominantly linalool and VPBs, which contribute to the sweet and honey-like smell of this tea.

## Conclusion

This study clearly shows that pre-harvest MeJA treatment has a high potential to improve the aroma of green, oolong, and black tea due to the release of volatile compounds and changes in amino acid levels induced by changes in the LOX and the terpenoid biosynthesis as well as the shikimate pathway. These changes occurred in fresh leaves, during key processing steps, and ultimately, affect the aroma of the final tea products as also confirmed by sensory evaluation. Further research is now required to unravel the underlying mechanisms leading to these changes in key compounds involved in conferring aroma and flavor in green, oolong, and black tea and should be complemented by absolute quantification of volatiles and related metabolites.

## Author Contributions

SB and ZL conceived and designed the research. JS drafted the manuscript and analyzed all data. JS, DX, DQ, and QP together carried out the whole experiments. All authors read and approved the manuscript.

## Conflict of Interest Statement

The authors declare that the research was conducted in the absence of any commercial or financial relationships that could be construed as a potential conflict of interest. The handling Editor is currently organizing a research topic with one of the authors, SB, and confirms the absence of any other collaboration.

## Abbreviations

GABA: gamma-aminobutyric acid
GC-MS: gas chromatography-mass spectrometry
MeJA: methyl jasmonate
PDMS: polydimethylsiloxane
SBSE: Stir bar sorptive extraction
VPBs: volatile phenylpropanoids/benzoids.
