# Supplementary material for: Methyl Jasmonate-Induced Changes of Flavor Profiles During the Processing of Green, Oolong, and Black Tea
Source: Front Plant Sci. 2019 Jun 14;10:781. doi: 10.3389/fpls.2019.00781 (PMC6587438; doi:10.3389/fpls.2019.00781)
Supplement: Supplementary file 1 [file Data_Sheet_1.docx]

Specificity of MeJA spraying and condition of the tea garden.

Weather condition: 16℃~20℃, no wind, cloudy.

Spraying time: 17:00

MeJA solution: preparing every 2 L 0.25% MeJA solution, then spraying on the surface of the tea plants till the forming of water drops. When the solution was finished, another 2L was prepared till the entire 2 hectares was sprayed with MeJA solution.
